# Fine-grained climate velocities reveal vulnerability of protected areas to climate change

**DOI:** 10.1038/s41598-020-58638-8

**Published:** 2020-02-03

**Authors:** Risto K. Heikkinen, Niko Leikola, Juha Aalto, Kaisu Aapala, Saija Kuusela, Miska Luoto, Raimo Virkkala

**Affiliations:** 1 Finnish Environment Institute, Biodiversity Centre, FI-00790 Helsinki, Finland; 2 Department of Geosciences and Geography, University of Helsinki, FI-00014 Helsinki, Finland; 3 Finnish Meteorological Institute, FI-00101 Helsinki, Finland

**Keywords:** Climate-change ecology, Climate-change impacts

## Abstract

Climate change velocity is an increasingly used metric to assess the broad-scale climatic exposure and climate change induced risks to terrestrial and marine ecosystems. However, the utility of this metric in conservation planning can be enhanced by determining the velocities of multiple climatic drivers in real protected area (PA) networks on ecologically relevant scales. Here we investigate the velocities of three key bioclimatic variables across a nation-wide reserve network, and the consequences of including fine-grained topoclimatic data in velocity assessments. Using 50-m resolution data describing present-day and future topoclimates, we assessed the velocities of growing degree days, the mean January temperature and climatic water balance in the Natura 2000 PA network in Finland. The high-velocity areas for the three climate variables differed drastically, indicating contrasting exposure risks in different PAs. The 50-m resolution climate data revealed more realistic estimates of climate velocities and more overlap between the present-day and future climate spaces in the PAs than the 1-km resolution data. Even so, the current temperature conditions were projected to disappear from almost all the studied PAs by the end of this century. Thus, in PA networks with only moderate topographic variation, far-reaching climate change induced ecological changes may be inevitable.

## Introduction

Measurements of the magnitude and geographic variation of climatic changes across the network of protected areas (PAs) provide relevant information for conservation planning, enabling the targeting of management in the PAs most at risk in the face of climate change^1–6^. One approach for assessing the climate-change-based risks is the climate change velocity, a metric which defines the speed and direction of climate shifts over a given area^4^. Although the majority of the climate velocity studies have been conducted in terrestrial environments, there is now an increasing amount of climate velocity research also addressing marine environments^4,7,8^.

Technically, climate velocity is a generic metric which nevertheless provides ecologically relevant information for climate-wise conservation planning^2,4,9^. Such information is particularly useful for identifying regions and PAs where climate conditions are changing most rapidly, exposing them to high rates of climate displacement^3^. Climate velocity has typically been used to assess the climatic risks for the persistence of species and populations^9^, but in cases where rapid changes in the climate affect ecological engineer and keystone species, profound impacts can be carried over to community structure and ecosystem functions^2^. Considering PAs as such, climate velocity assessments can be used to identify PAs which face substantial difficulties in retaining ecological conditions that promote present-day biodiversity. Moreover, climate velocity analyses are important in regions which would need new stepping-stone conservation areas to support species movements to complement the PA network, or conversely, to detect PAs with particularly low climate velocities which could provide potential climate refugia for local populations^3,4,6,10^.

However, different methodological aspects may markedly affect velocity measures and subsequent risk assessments in the PAs. First, in previous studies climate velocity is often assessed using only one variable, particularly the mean annual air temperature on land^11,12^, and in marine environments the mean sea surface temperature of the ocean^7,8^. Such a focus on single variables provides a limited understanding of climate-based risks for biodiversity^11,13,14^. Velocity studies that include several variables have mainly used multivariate climate gradients constructed using a principal components analysis (PCA)^6,9^. However, while PCA-axes effectively describe the overall climate space, they may obscure the interpretation of impacts of separate key drivers^1,4^. An alternative approach used in a few recent studies is to assess the climatic exposure of different regions using multiple individual variables^9,12,13^; these studies have reported notable spatio-temporal variations between the velocities of different climate variables. However, these assessments have rarely been calculated for real regional and national reserve networks although they would enable the detection of potentially divergent future threats to biodiversity in different PAs^9,12,13^.

Second, earlier cross-scale studies have shown that velocity values tend to decrease towards finer resolutions^9,11^. However, these studies have been restricted to the domains of the mesoclimate (resolutions of 1–100 km) or macroclimate (>100 km scales)^2^, and none of the velocity resolution comparisons in terrestrial environments have examined the impacts of truly fine-grained (<100 m) climatic conditions created by local variation in topography, i.e. the topoclimate^2,9,11^. The same predominance of meso- and macroclimates is evident also in single-scale velocity studies which – except for Liang *et al*.^15^ – have employed climate data at a resolution of 800 m or coarser^2,4,9,16^. This overlook of topoclimatic patterns in the velocity assessments of PAs may lead to biased exposure assessments especially in rugged terrain^3,11,17^, as well as a limited ability to detect sites decoupled from the regional climate^18–20^ and insufficient understanding of the degree of the overlap between present-day and future climate conditions in PAs^2^. A similar strong bias towards meso- and broad-scale velocity studies is evident also in marine environments, although substantial fine-scale climate change impacts and spatio-temporal climate refuges exist in the oceans^21^.

Here, we apply a climate velocity approach to provide the first assessment of the climatic exposure risks across a national PA network based on very fine-grained velocities of three established drivers of high latitude terrestrial biodiversity^11,22^, measured on a spatial scale which reflects the local impacts of topoclimate. Our variables describe the future topoclimatic patterns both for winter and summer air temperatures, as well as for the climatic water balance^23^. The study area stretches over 1,000 km from hemiboreal deciduous forests via boreal coniferous forests to treeless tundra, representing a region where climate change is faster than the global average^5^. Our study system is the 1,778 protected areas included in the Natura 2000 network and situated in the mainland Finland. From this PA network we dissected 5,068 physically separate Natura 2000 PA polygons (hereafter referred to as ‘Natura PAs’ or simply ‘PAs’) for the purposes of this study using a number of selection criteria (see Methods). Natura 2000 network is a part of the largest network of systematically selected protected areas in the world covering the most valuable species and habitats in 28 European Union countries^24,25^.

## Methods

### General approach

To produce fine-grain climate velocity measures, we modelled monthly temperature and precipitation data averaged for the period from 1981 to 2010 at a resolution of 50-m, incorporating the physiographic effects of solar radiation and cold-air pooling. Based on these data, we calculated estimates for the annual temperature sum above 5 °C (growing degree days, GDD, °C), the mean January temperature (T_Jan_, °C) and the annual climatic water balance (WAB, the difference between annual precipitation and potential evapotranspiration; mm) for our 50-m grid system covering Finland and the adjacent areas (Supplementary Fig. 1S). To investigate how the data resolution affected the velocity measures, 50-m resolution climate surfaces of the three variables were spatially mean-aggregated to a 1-km resolution. For both resolutions, similar future climate surfaces were produced using an ensemble of 23 global climate models from the CMIP5 archives for the years 2070–2099 and the three RCP scenarios (RCP2.6, RCP4.5 and RCP8.5)^26^. Then using climate-analog approach^3,4,9^, we measured climate velocities for each of the 50-m and 1-km grid cells based on the distance between climatically similar cells under the baseline and the future climates. In the final step, velocity values were averaged for the focal 5,068 Natura 2000 PA sites to examine their climatic exposure and to assess the overlap between the current and future range in topoclimatic conditions in the PAs.

### High-resolution climate data

We developed monthly average temperatures (1981–2010) over the study domain at a spatial resolution of 50 × 50 m by building topoclimatic models based on data sourced from 313 meteorological stations (European Climate Assessment and Dataset [ECA&D])^27^. The station network and modeling domain covered Finland with an additional 100 km buffer, but it was also extended to cover parts of northern Sweden and Norway for areas >66.5°N (Supplementary Figs. 1S and 2S). This was done because under the current climate changes, future climate spaces similar to the present climate in Finland may be found outside the country borders. Capturing the analogical climate space in areas beyond the country borders is essential in order to avoid developing a large number of velocity values deemed as infinite or unknown. This is especially important in the approach applied in the study, i.e. measuring climate change velocity with the climate-analog velocity approach^4,9^. In total, our modeling domain constitutes nearly 50 million grid cells.

The methodology to produce the average air temperature data is fully described in Aalto *et al*.^18^, and thus only briefly explained here. For temperature modelling we applied generalized additive modeling (GAM) as implemented in the computer software R-package mgcv version 1.8–7^28,29^, utilising variables of geographical location (latitude and longitude, included as an anisotropic interaction), topography (elevation, potential incoming solar radiation, relative elevation) and water cover (sea and lake proximity). A leave-one-out cross-validation suggested that our modelled monthly average air temperatures agreed well with the observations, with the root mean squared error (RMSE) ranging from 0.37 °C (July) to 1.49 °C (January; Supplementary Fig. 3S).

To produce gridded average annual precipitation data (1981–2010), we used global kriging interpolation based on the data from 343 rain gauges obtained from the ECA&D dataset (Supplementary Fig. 2S), geographical location, topography (elevation and eastness index) and proximity to the sea. Kriging interpolation was done using R package gstat version 1.1–0^30^. The eastness index was obtained from a sine-transforming aspect raster surface calculated from a 50 m × 50 m digital elevation model to capture the effect of westerly winds (the prevailing wind direction in the region) on the accumulated precipitation on windward slopes in mountainous areas. To ease the computational burden of the kriging procedure, the gridding was run at a resolution of 500 × 500 m. This was also justified as we expected the annual average precipitation to mainly reflect large scale orographic features, and thus not to significantly vary across short geographical distances. After this, the gridded annual precipitation was bilinearly interpolated into the same 50 × 50 m resolution as the air temperature data. A leave-one-out cross-validation was conducted over the gauge data and indicated a reasonable agreement between the measured and interpolated average annual precipitation sum with an RMSE of ca. 93 mm (Supplementary Fig. 4S).

### Bioclimatic variables

**Figure 1:**
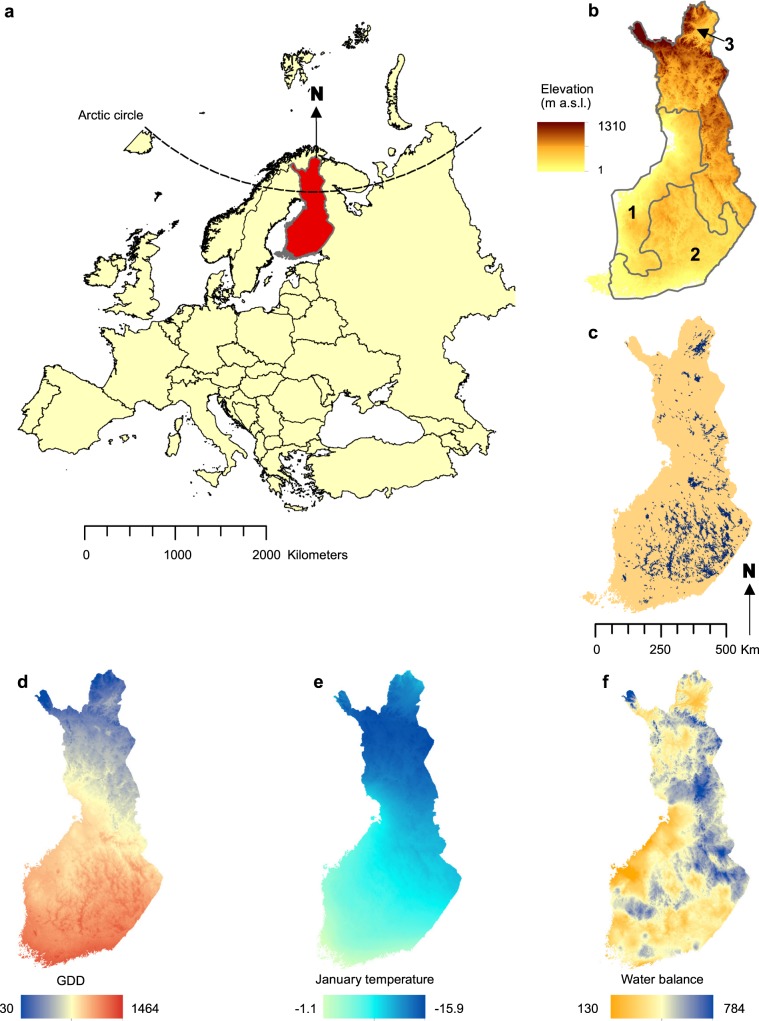
The study area and geographic variation of five environmental variables. (**a**) Location of the study area in northern Europe. (**b**) Elevation (m a.s.l.), and three main topographic relief regions in mainland Finland (1 = flatlands; 2 = gently undulating hilly terrain; 3 = rugged terrain with notable variation in elevation), **(c**) lakes, (**d**) growing degree days (base temperature 5 °C; GDD), (**e**) mean January temperature (°C), (**f**) climatic water balance (mm, difference between annual precipitation sum and potential evapotranspiration). (**d–f**) Represent average conditions over 1981–2010, modelled at a resolution of 50 × 50 m.

Three bioclimatic variables: the annual temperature sum indicating the accumulated warmth (growing degree days, GDD, °C); the mean January air temperature (T_Jan_, °C); and the climatic water balance (WAB, mm) were calculated from the high-resolution gridded climate data (Fig. 1). We focused on these three bioclimatic variables because several earlier species – climate modelling studies have employed same variables and demonstrated their ecological relevance to a large number of habitats and species from different taxonomical groups inhabiting northern environments^22,31–36^. Taken together, these three variables effectively complement each other by providing estimations of winter cold, seasonal warmth and moisture availability which are among the key drivers of biodiversity in our study region. For example, GDD determines the reach of maturity, blooming and completion of life cycle in plant species and the development threshold of insect larvae, the mean temperature of January affects the overwintering survival of species, and the climatic water balance determines the moisture availability for both plant and animal species and the level of drought stress.

GDD represents the effective temperature sum above the base temperature of 5 °C^37^:

$$GDD5={\sum }_{i}^{n}({T}_{i}-{T}_{b}),\,if\,{T}_{i}-{T}_{b} > 5$$

where *T*_*i*_ denotes the mean temperature at day *i*, *T*_*b*_ represents the base temperature, and *n* is the length of the summation period. Since the daily air temperature data was not available, we estimated the GDD using monthly data following Araújo and Luoto^38^. The WAB is the difference between the total annual precipitation sum and the potential evapotranspiration (PET), which was estimated from the monthly air temperatures following Skov and Svenning^39^:

$$PET=58.93\times {T}_{above{0}^{^\circ }C}/12$$

### Coarse resolution climate data

To investigate how the climate data resolution affects the derived climate velocity measures, the high-resolution (50 × 50 m) climate surfaces were spatially aggregated onto a 1 × 1 km resolution grid using an areal mean function. Additionally, although spatially comprehensive, our study domain does not cover parts of western Russia which are adjacent to NE Finland, and which could be a direction for the escaping climates in the future^40^. Because data and computational constraints prevented us from realistically expanding the topoclimate modeling domain further eastward from the 100 km buffer zone depicted in Supplementary Fig. 1S, the E-OBS data set (version 17.0; 25 km × 25 km)^41^ covering the areas of ca. 30–50°E were employed, where necessary, to complement the climate velocity analyses for T_Jan_.

### Global climate model data

To develop data on future climates, we used the climate projections for the twenty-first century based on an ensemble of 23 global climate models (GCMs), as available from the Coupled Model Intercomparison Project phase 5 archives^26^. These data were processed to represent the predicted averaged changes in mean temperature and precipitation (with respect to the baseline 1981–2010) over the period of 2070–2099, and three RCP scenarios (RCP2.6, RCP4.5 and RCP8.5)^42^. The climate model data depicting the predicted change in mean temperatures and precipitation with respect to the baseline climate were bilinearly interpolated onto a matching resolution of 50 × 50 m, and the change predicted by the GCMs was added to the spatially detailed baseline climate data. After this, the bioclimatic variables were recalculated for each RCP scenario to allow the subsequent calculation of climate change velocities and measuring the overlap of the current and future climate ranges within the PAs.

### Climate velocity metrics

We calculated the climate change velocities for the three studied bioclimatic variables, GDD, T_Jan_ and WAB, using the approach developed by Hamann *et al*.^9^, referred to as the distance-based velocity or climate-analog velocity^4^. In this approach, climate-velocity metrics are calculated by measuring the distance between present-day locations with certain climatic conditions and their future climate analogues, divided by the number of years between the two points in time^4^. We calculated the climate-analog velocities both for the 50-m resolution grid and the 1-km resolution grid climate data by measuring the distance between climatically similar grid cells for the present and future climates determined by the three climate scenarios, RCP2.6, RCP4.5 and RCP8.5.

Following Hamann *et al*.^9^, we selected the boundary values for the classes of bioclimatic variables so that the climatically matching grid cells were defined by setting the within-class range to be as small as possible while, at the same time, avoiding artefactual extreme precision, which could produce unrealistic sporadic patterns in the velocity. To achieve this target, we tested 2–3 different settings for each of the three climate variables and carried out a literature search for earlier class definitions applied to corresponding climate variables. Based on this, the following within-class ranges were selected for the consequent climate velocity analysis: GDD, within-class range 50 °C; T_Jan_, within-class range 0.5 °C; WAB, within-class range 50 mm. The present-day and future climate surfaces of the three climate scenarios were then reclassified by assigning the continuous climate values in each of the 50-m grid cells in one of the 51 GDD, 60 T_Jan_, and 55 WAB categories covering the overall current and future range in the climate space of these variables. This enabled the execution of the search of the minimum distances between grid cells with similar present-day and future GDD/T_Jan_/WAB climates. In technical terms, the search was carried out using the ArcGIS software (Desktop 10.5.1.) by employing the Euclidean distance function. The recommendation by Hamann *et al*.^9^ on the “use of gridded data with as high resolution as is computationally feasible and justifiable based on the precision of interpolated climate grids” was achieved in our study by building fine-grained 50-m resolution topoclimatic models, i.e. we used an approach whose methodology and accuracy had been tested in our previous work^18^.

The resulting measures of the climate velocities for the three climate variables were employed in a series of subsequent analyses. The high-velocity areas (‘velocity hotspots’) of the three climate variables were visually compared with each other based on maps showing their 50-m resolution velocities across the whole of mainland Finland.

### Exposure of the protected areas

From the 1,778 protected areas included the Natura 2000 network in mainland Finland we selected 5,068 physically separate Natura 2000 sites (polygons) for this study. Following Nila and Hossein^25^, we refer to these 5,068 sites as ‘Natura 2000 PAs’, or simply as ‘PAs’. Throughout the European Union, the Natura 2000 network aims to cover the most valuable species and habitats in the 28 member countries (see https://ec.europa.eu/environment/nature/natura2000/index_en.htm). The selection of Natura 2000 PAs was done so that first we dissected each physically separate Natura 2000 PA (i.e. Natura 2000 polygons physically disconnected from other Natura 2000 polygons; e.g. a Natura 2000 area consisting of 5 separate polygons were treated as five individual PAs), then we measured the area of each PA and selected those with a cover of 2 hectares or more. Another selection rule was that from the islands situated close to the coastline of mainland Finland, only PAs on larger islands were included (to avoid the inclusion of smaller islands and islets abundantly surrounded by water areas). For the 5,068 Natura 2000 PAs, we defined the high-velocity PAs (velocity hotspots) as the top 5% showing the highest velocity values. These top 5% high-velocity PAs consisted of 253 sites located in mainland Finland with the highest velocities out of the total of 5,068 PAs. These were assessed separately for the three climate variables, GDD, T_Jan_ and WAB, by calculating the mean of the corresponding velocities in the 50-m grid cells included in a given Natura 2000 PA. Based on these data, we assessed in how many of the PAs the velocity hotspots overlapped for two or three variables in the three RCP scenarios (RCP2.6, RCP4.5 and RCP8.5).

### Comparison of 50-m and 1-km velocities

The comparisons of the velocity values at the 50-m and the 1-km resolutions were done using only data for the GDD. This was because the velocity patterns for the WAB were rather localised and complex, and because the disappearing climate space in the mean January temperatures would complicate the resolution comparisons in the PAs located in N Finland. The comparisons of the GDD 50-m vs. 1-km resolution velocities in the 5,068 PAs were done in two ways. First, we compared the *absolute* values of 50-m and 1-km GDD velocity values in each of the 5,068 PAs. For these comparisons, mainland Finland was divided into three relief regions on the basis of the general elevational and topographic features of the terrain (Fig. 1): (i) Relief region 1: flatlands with mostly even terrain (in this region, 10 × 10 km grid squares typically show height differences below 50 m), (ii) Relief region 2: with hilly, undulating terrain varying in height (with typically 50–200 m height differences in 10 × 10 km squares), and (iii) Relief region 3: with rugged terrain and deep valleys or high steeply sloped fell areas (with typically over 200 m height differences in the 10 × 10 km squares). Per-PA comparisons of the absolute differences in the two velocity values were made to assess the number of cases where the 50-m resolution GDD velocity was higher than the 1-km resolution velocity, and vice versa, and to examine the significance of these differences using a paired t-test. These comparisons and tests were done separately for each relief region and each of the three RCPs.

Second, we examined the *relative* differences between the 50-m and 1-km GDD velocities in the 5,068 PAs. This was done in order to take into account the fact that similar absolute differences in the two velocity values may have a different ecological importance when occurring at different overall levels (e.g. a difference of 0.2 in the velocities very likely matters more between 0.1 and 0.3 than between 2.1 and 2.3). Then, we determined the top 5% of the PAs with largest relative differences between the 50-m resolution and the 1-km resolution velocities and examined how they were divided in the three main relief regions of Finland.

Next, we used generalized linear models (GLMs)^43^ to test the importance of the relief region, size of the Natura 2000 PA, within-PA elevation range and climate change scenario in explaining the relative differences between the 50-m and 1-km GDD velocities. We fitted a full GLM model where all four explanatory variables were considered at the same time. In this model, relief region was treated as ordinal variables with three levels, the size of the Natura 2000 PA and within-PA elevation range both as log-transformed continuous variables, while the forth variable, climate scenario (RCP2.6, RCP4.5 and RCP8.5), was treated as a categorical factor. Our main interest was in the first three variables (the relief region, the size of the PA and the elevation range within the PA). For these variables, we calculated their effect sizes based on the range between their predicted minimum and maximum values in the observation data while controlling for the influence of other predictors by fixing them at their mean values^44^.

### Overlapping of present-day and future climate spaces in Pas

In addition to the climate velocity analysis, we also examined the degree of overlap between the present-day range and projected future range of the three climate variables in each of the 5,068 Natura 2000 PAs. In order to assess the impact of the data resolution, these analyses were carried out using both the 50-m and the 1-km resolution climate data. Here, we examined whether the fine-grained topoclimate data showed more overlapping between the present-day and the future ranges than the mesoscale 1-km climate data in the PAs. Moreover, in cases where there were not any overlapping parts in the climate spaces, we assessed whether the estimated size of the gap between the present-day and the future ranges depended on the resolution of the climate data. All these assessments were done three times by comparing the present-day within-PA climatic ranges with the projected ranges under the three climate scenarios, RCP2.6, RCP4.5 and RCP8.5. It should be noted that the future within-PA ranges for the GDD and T_Jan_ were always either overlapping with the present-day range or located above it, but for the WAB values they overlapped with the present-day range or were located either above or below it (i.e. indicating locally varying, contrasting future changes).

## Results

**Figure 2:**
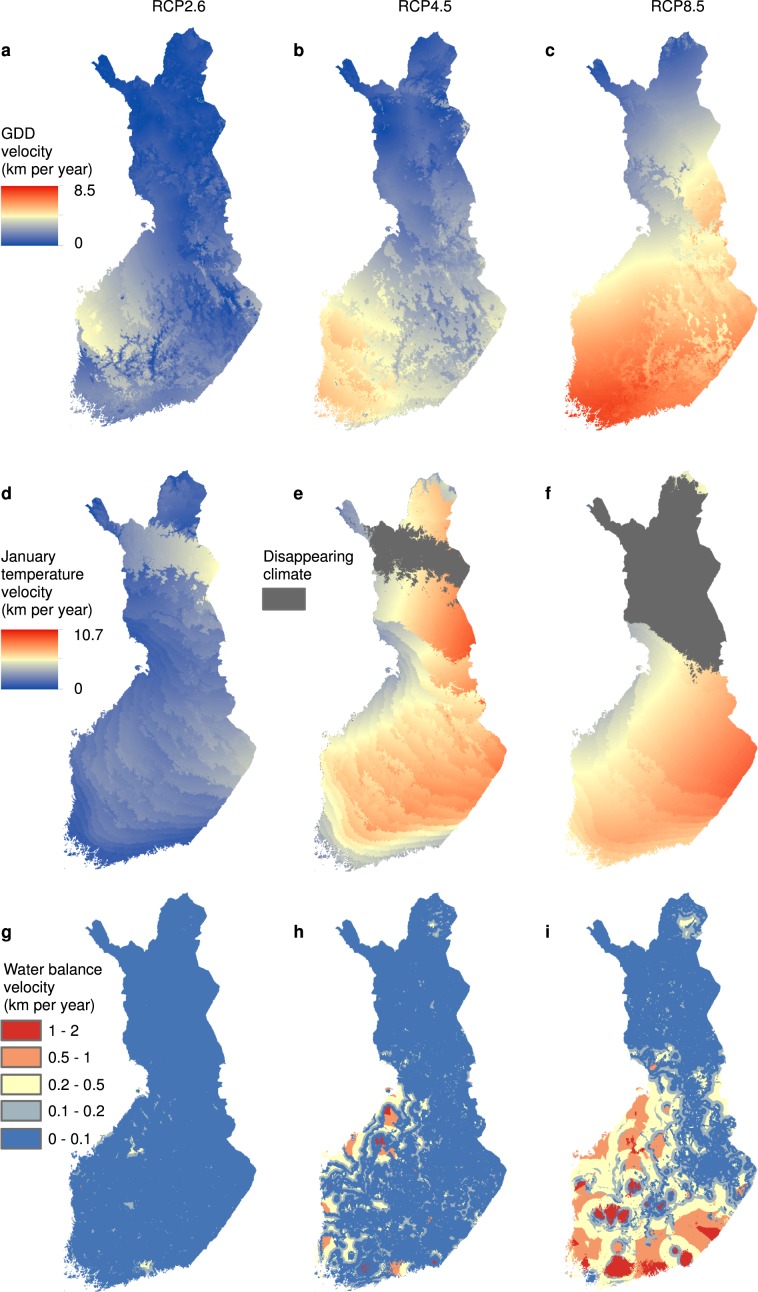
Fine-grained climate-analog velocities of three climate variables in Finland. (**a–c**) Growing degree days (GDD), (**d–f**) mean January temperature, (**g–i**) climatic water balance. The velocities are calculated as the minimum Euclidean distance between the closest climatically similar location in the current climate and in the three future climates (**a**,**d**,**g** – RCP2.6; **b**,**e**,**h** – RCP4.5; **c**,**f**,**i** – RCP8.5), divided by the time separating the two periods, 1981–2010 and 2070–2099. The velocities were measured for all 50 × 50 m grid cells occurring in mainland Finland by extending, where required, the search of climate analogues beyond country borders (see Supplementary Fig. 1S). Note that, despite this, (**e,f**) suggest disappearing climate conditions.

The three climate variables showed complex spatial patterns of fine-grained velocities with contrasting high-velocity areas (i.e. the top 5% areas with the highest velocities) and different rates of velocity (Fig. 2). For all climate variables, the velocities increased towards the most severe climate scenario, RCP8.5. On average, they were the lowest for the WAB values (Fig. 2g–i) and highest for the T_Jan_ values (Fig. 2d–f). For the GDD (Fig. 2a–c), the highest velocities occurred in Southwest Finland, while for T_Jan_ the highest velocities, and areas of completely disappearing winter thermal conditions, occurred in North Finland (Fig. 2e,f). The spatial patterns of the WAB velocities are complex, with mainly low velocities accompanied by scattered regions of higher velocities. In our PAs, the three climate variables very rarely coincided in high-velocity areas, ranging from a 15.8% overlap between the GDD and WAB to zero overlap between GDD and T_Jan_ hotpots (Supplementary Table 1S).

**Figure 3:**
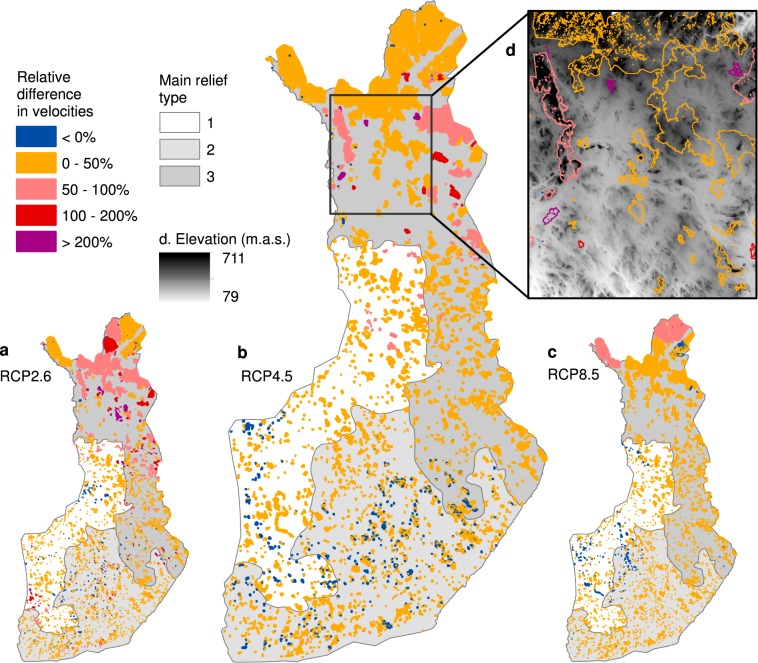
Relative difference between 50-m resolution and 1-km resolution GDD velocity values in the protected areas (PAs) included in the Natura 2000 network (n = 5,068). The results are shown for three relief regions (1 = flatlands; 2 = gently undulating hilly terrain; 3 = rugged terrain) and three climate scenarios; (**a**) RCP2.6; (**b**) RCP4.5; (**c**) RCP8.5. Relative differences in velocities larger than zero indicate PAs where 1-km resolution velocities are larger than 50-m resolution velocities, and values which are smaller than zero indicate PAs where the 50-m resolution velocities are larger than 1-km resolution velocities. (**d**) Shows a zoomed-in example area in topographically heterogeneous, rugged terrain in North Finland.

Due to the localised patterns of the WAB velocity and disappearing climate space in T_Jan_, we focused our comparisons on the GDD velocities from the 5,068 PAs (Fig. 3). A comparison of the *absolute* differences between the velocities from the two resolutions shows that the mean GDD velocities were consistently higher at the 1-km resolution in all relief regions and under all climate scenarios (Supplementary Table 2S). Moreover, the proportion of PAs where the 1-km velocity was higher than 50-m velocity was >81% in all comparisons, and in the most topographically rugged relief region (region 3) it was >90%.

The largest *relative* differences between the 50-m and 1-km resolution velocities in the PAs also occurred systematically more often in region 3 (Fig. 3, Supplementary Table 3S) although within-region variation also exists (Fig. 3d). The full GLM model showed that all three key considered variables (the relief region, size of the PA and within-PA elevation range) contributed significantly (p < 0.001) to the variation in the relative differences between the two velocity values (Supplementary Table 4S). However, the t-values from the GLM model and the effect size of our three explanatory variables showed that the relief region and within-PA elevation range were the two most important, with their order of importance switching between the two test results. Considering the effect of climate scenarios, the relative differences between the 50-m and 1-km velocities were the highest in RCP2.6 and lowest in RCP8.5 (Fig. 3), suggesting that the buffering potential of the topoclimate decreases with increased climate warming.

**Figure 4:**
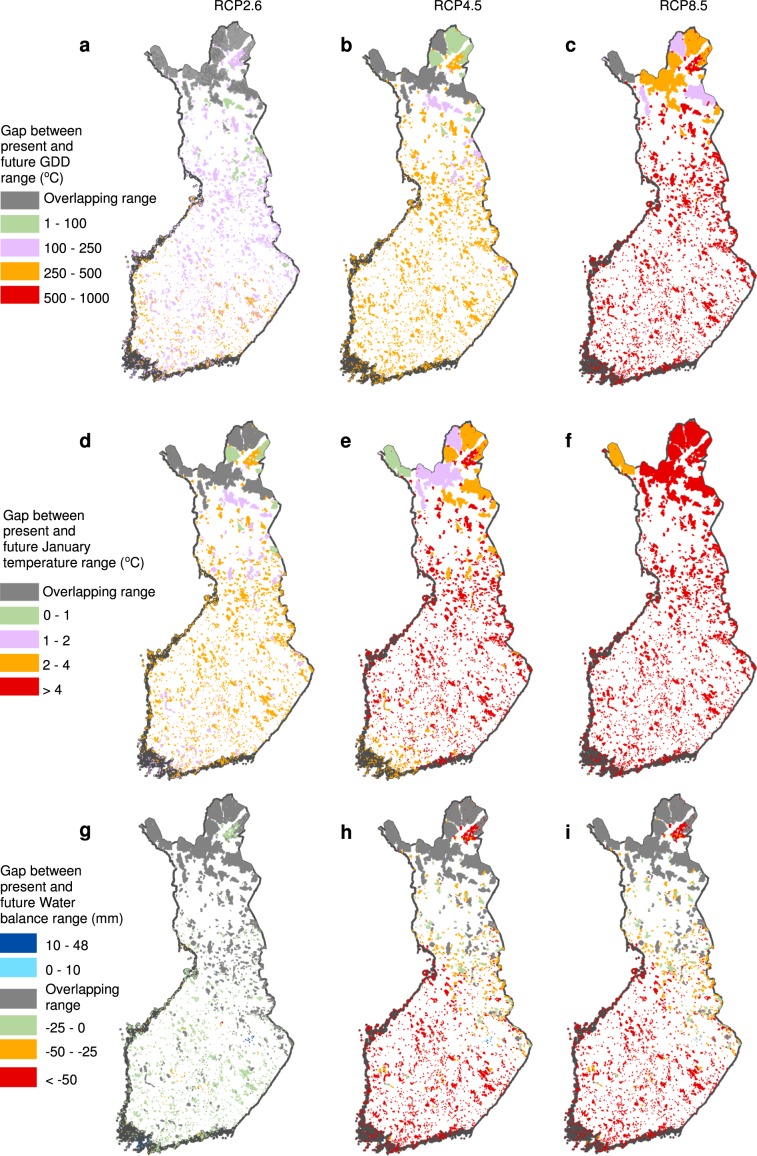
The gap between the present-day and projected future fine-grained ranges for three climate variables in 5,068 Natura 2000 PAs. (**a–c**) Growing degree days (GDD, °C); **(d–f**) mean January temperature (°C); (**g–i**) climatic water balance (mm). The fine-grained (50-m resolution) within-PA future ranges of climate variables are based on RCP2.6 (**a**,**d**,**g**), RCP4.5 (**b**,**e**,**h**) and RCP8.5 (**c**,**f**,**i**). For GDD and the January temperature, the future range either overlaps with the current range or is fully separated above it (indicating a trend towards warmer conditions), whereas for the climatic water balance, the future range overlaps with the current range, or is fully separated either above (positive values, indicating increasingly moist conditions) or below (negative values, indicating increasingly dry conditions) it.

The 50-m resolution climate data suggests that present-day and future climate spaces overlap in the studied PAs more often than the 1-km data show (Supplementary Table 5S). The degree of climatic space overlap varies greatly between the climate variables (Fig. 4). Between 76 and 564 PAs at 1-km resolution, and 103 and 823 PAs at 50-m resolution, respectively, are projected to have partly similar water balance conditions as they do at the present, but there is very little overlap between the present-day and future ranges of GDD and T_Jan_. Similarly, fine-grained topoclimate data on the T_Jan_ overlaps only in the climate scenario RCP2.6 (Supplementary Table 5S). The commonness of the overlap decreases with the increasing severity of the climate scenario, e.g. in GDD, overlap occurs under RCP8.5 in only one PA with an elevation range >1,000 m (Figs. 1b and 4). In PAs with no overlap, the gaps between the current and future conditions are on average larger at the 1-km than the 50-m resolution (Supplementary Table 6S) and increase towards RCP8.5 (Supplementary Figs. 5S–7S). In GDD and T_Jan_, the future range is projected to mainly occur above the current range, whereas for WAB it overlaps with the current range, or, in most cases, occurs below it, suggesting a trend towards drier conditions (Supplementary Table 6S).

## Discussion

Earlier studies have shown that velocity assessments based solely on one climate variable provide limited understanding of exposure risks^11,13^, and that velocities of annual and seasonal temperature and precipitation variables may differ considerably^12,13,45^. Here, we have shown that fine-grained topoclimate velocity patterns and high-velocity areas can also diverge drastically between moisture and temperature variables, as well as between summer and winter variables. In agreement with earlier findings^13,15^, the velocities for temperature variables are higher than for the water balance, peaking at >10 km/year for T_Jan_ (Fig. 2).

The fine-grained high-velocity areas of the three climate variables rarely coincided in our PAs, suggesting that ecologically different species may face climate change-induced exposure risks very differently. The velocity of GDD peaks in the flatland areas in Southwest Finland, where already modest warming can cause large geographic climate displacement^25^. These findings have alarming implications for conservation planning since this region is characterised by a mixture of high species richness, sparse PA networks and a human-modified matrix between the PAs. In addition, notable climate displacement in GDD takes place also in the PAs situated in larger islands on the SW coast of Finland, especially under RCP 4.5 and RCP8.5 projections (Fig. 2). This indicates that species populations occurring in these islands have very limited space to track changes in climate even though many of the islands show a greater variation in elevation than the coastal flatlands. Successful tracking of climatically suitable space by species is further restricted by the large water areas especially between outer archipelago PAs and the mainland, making the island populations highly vulnerable to climate change.

Equally importantly, under RCP4.5 and RCP8.5 projections, North Finland is widely projected to lose the coldest January climates, with potentially devastating impacts on species favouring cold winters^45,46^. In agreement with the findings from Australia^12^, these are not solely poleward patterns. Because winters are relatively warmer in coastal rather than continental areas, the coldest winter climates will move several hundreds of kilometres eastwards, towards Siberia. In contrast to the temperature velocities, fine-grained water balance velocities are milder and spatially sporadic. This irregularity probably emerges partly from the impacts of the coastline and inland waterbodies on the local rainfall patterns^47^, and partly from the difficulties in accurate modeling of fine-grained precipitation-related variables^48^, calling for caution in their use in climate exposure assessments.

From the four ‘climate domains’, macroclimate (>10 km), mesoclimate (1–10 km), topoclimate (10m-1km) and microclimate (<50 m)^2,20^, velocity studies have heavily focused on the macroclimate^4,5^ and mesoclimate scales^11,16^. This overlook of the topoclimate and microclimate has hindered the detection of climatically poorly coupled localities^2,18,20^ which may provide potential holdouts for species to resist climate change^4,10,17,19,49^. Here, using 50-m resolution climate data, we were able to examine the impacts of the topoclimate, reflecting the variation in incoming solar radiation and cold air drainage, on the climate velocity estimates. Similarly to broader scales^9,11^, the GDD velocities were regularly lower at finer resolutions (50-m) than on a coarser scale (1-km). Consequently, climatically similar locations occurred, depending on the climate scenario and local topography, on average 12–45 km nearer in the landscape than the 1-km climate data suggest. The largest relative differences between the 50-m and 1-km velocities occurred more often in the most topographically rugged regions including gorges and high fells^2,9,17^. However, within-region variation also occurred, suggesting that the consideration of the topoclimate alters the velocity estimates most notably in PAs which are embedded in the lowlands of an otherwise topographically variable landscape (Fig. 3d).

Comparing the fine-grained current and projected future ranges of climate variables within the PAs enables the assessment of whether employing topoclimate data alters the broadscale estimates of the degree or the lack of overlap between the future coolest locations and the present-day warmest sites^2^. Our results show that the contemporary and future topoclimate ranges of both temperature and water balance variables indeed overlap more often in PAs than the 1-km climate data suggests. This indicates the potential for topoclimatically deviant sites to support species’ extended persistence^4,10,17,49^. Thus, the fine-grained topoclimate variation has the potential to provide significant buffering against climate change in topographically rugged PAs, which is not readily visible in mesoscale velocities^2^.

However, in our case, the potential for topoclimatic buffering appears to be limited. For summer and winter temperature variables, the within-PA overlap is very modest, especially for January temperatures (Fig. 4d–f). Thus, our results reveal similar, substantial, within-PA turnover of climate space, as the earlier global^16^ and regional^2^ velocity studies, where only the largest PAs were projected to experience temperatures which would be similar to today. Our finding that topoclimatic variation may have only a limited buffering potential has significant implications for conservation planning in many of the European Natura 2000 areas with modest elevation ranges, especially in the lowlands of Europe. Moreover, PAs located in flatlands around the circumboreal region, e.g. in North Russia, face the same risks, further boosted by the strong changes in the climate. It should be acknowledged that gathering even finer resolution climate data (i.e. microclimate data) might reveal convergent environments which are not visible in the topoclimate data, such as shaded gorges and ravine forests with high canopy cover, which could maintain climatic microrefugia, and help species persistence under climate warming^19,20,50^. However, constructing comprehensive microclimatic data across a nation-wide PA network would be a technically very demanding exercise^10,50,51^, and is thus not yet feasible.

Examining fine-grained climate velocities and within-PA variability of multiple key variables increases the understanding of the potential threats to species which are differently sensitive to the climate^11,13,45^. Our analysis reveals a modest overlap between the current and future fine-grained temperature conditions even in the PAs with large elevation ranges. Furthermore, the high velocities detected here re-emphasise the arguments that adaptive conservation management and preparing for ecological change are crucial directions for climate-wise conservation planning^1,52^. Increasing attention needs to be paid to establishing well-connected groups of topographically heterogeneous PAs, and conserving habitats with deviant microclimates that provide holdouts for extended species persistence, and stepping-stones for dispersal^2,10^. In the identification of such holdouts and stepping-stones, the long-neglected issue in climate velocity research, topoclimatic variation, can play an integral role.

## Supplementary information

Supplementary Information .

## Data Availability

The data which support the findings of this study are available from the corresponding author upon request.
