# Supplementary material for: Fine-grained climate velocities reveal vulnerability of protected areas to climate change
Source: Sci Rep. 2020 Feb 3;10:1678. doi: 10.1038/s41598-020-58638-8 (PMC6997200; doi:10.1038/s41598-020-58638-8)
Supplement: Supplementary file 1 — Supplementary Information . [file 41598_2020_58638_MOESM1_ESM.pdf]

Fine-grained climate velocities reveal vulnerability of protected areas to climate change

Risto K. Heikkinen, Niko Leikola, Juha Aalto, Kaisu Aapala, Saija Kuusela, Miska Luoto, Raimo Virkkala

Supplementary Material

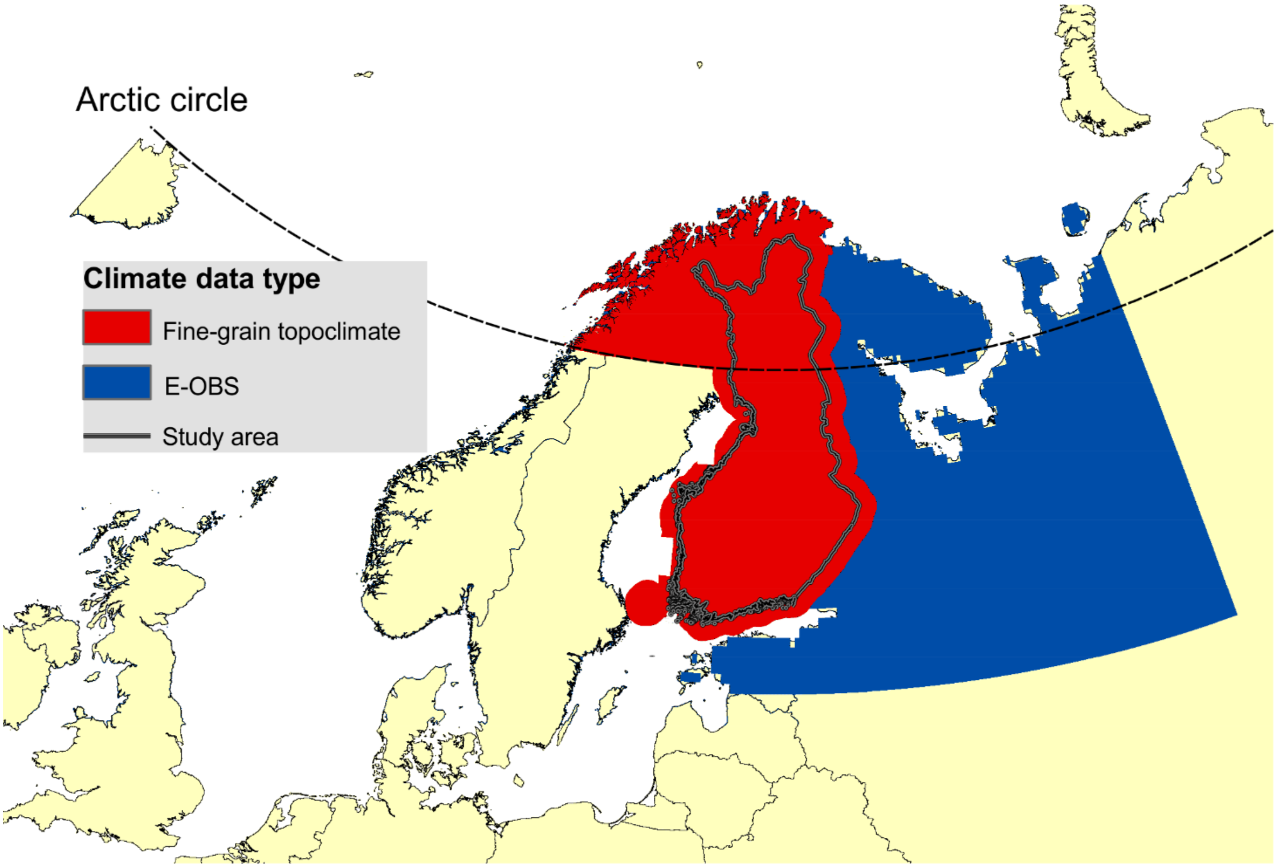

Supplementary Fig. 1S. Map of the climate data sources used in the study. Two different climate data sets were employed to develop the climate-analog velocity measures for the focal study area, i.e. mainland Finland, using a grid system with a resolution of 50 x 50 m. Velocity measures were developed for three bioclimatic variables: 1) the annual temperature sum above 5 °C indicating the accumulated warmth (growing degree days, ‘GDD’; measurement unit °C), 2) the mean temperature in January (°C), and 3) the annual climatic water balance (the difference between annual precipitation and potential evapotranspiration; mm). The primary climate data employed in the study, marked in red on the map, were the fine-grained topoclimate data. These data were developed based on monthly climatic averages for the years 1981-2010 by modelling the spatial variation in air temperatures and precipitation over the focal study area (mainland Finland)

and its neighbouring areas in Sweden, Norway and Russia at a spatial resolution of 50 × 50 m (see Aalto et al. 2017; Methods; Supplementary Fig. 5S). Our topoclimate models were based on data extracted from 313 meteorological stations located in Finland, Sweden and Norway. The projections from the models were developed to cover not only Finland but also an additional 100 km buffer area east of Finland, in Russia, and furthermore, large areas north of Finland, to cover the mountainous parts of northern Sweden and Norway. These areas situated beyond the country borders enabled the search of analogous climate spaces outside Finland.

The search for climatically analogous areas with respect to the mean January temperature showed that the future climates that correspond to the projected winter conditions of the RCP4.5 and RCP8.5 scenarios no longer offer analogous, equally cold winter temperatures as there are in the present-day topoclimate domain in certain areas of Finnish Lapland. Thus, we extended the search for climatically analogous areas several hundred kilometres east of Finland into Russia, using the broadscale mean January temperature data extracted from E-OBS Temperature and Precipitation Datasets, marked in blue on the map (Cornes et al. 2018). These data are produced at a resolution of 25 km. Using the 50-m grid system extended to Russia and the bilinear resampling procedure in ArcMap, we resampled these data to match the 50-m grid system employed in our study. However, as these data have rather limited potential to show the topoclimatic variation in the northwest areas of Russia, we used them only to supplement the velocity measurements for the January temperature for RCP4.5 and RCP8.5 in the north of Finland, when needed.

References

Aalto, J., Riihimäki, H., Meineri, E., Hylander, K. & Luoto, M. 2017: Revealing topoclimatic heterogeneity using meteorological station data. Int. J Clim., **37**, 544-556.

Cornes, R., G. van der Schrier, E.J.M. van den Besselaar, and P.D. Jones. 2018: An Ensemble Version of the E-OBS Temperature and Precipitation Datasets, J. Geophys. Res. Atmos., **123,** 9391-9409.

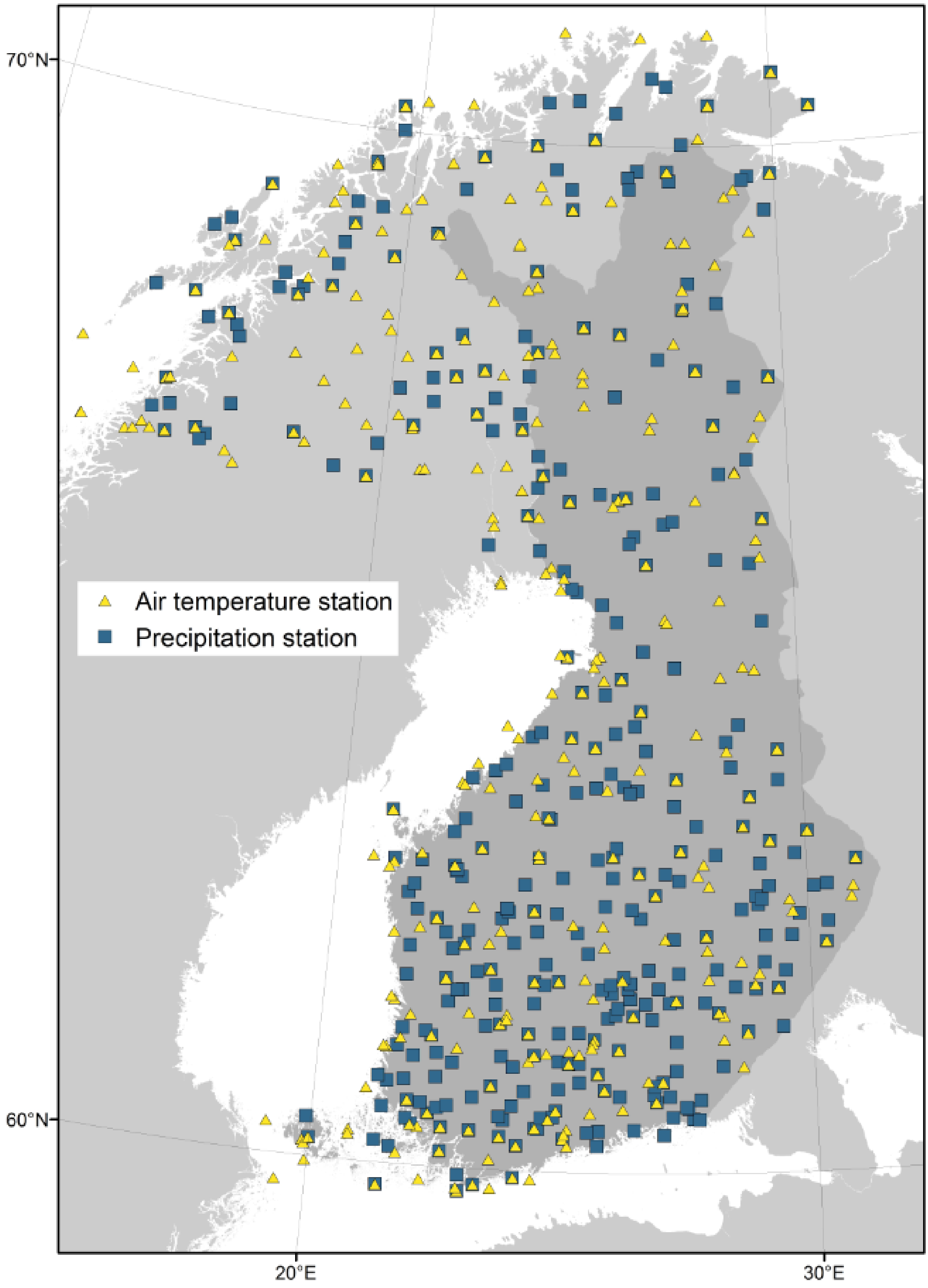

Supplementary Fig. 2S. The station network used for predicting the average air temperature (n = 313) and precipitation (n = 343) conditions over the study area.

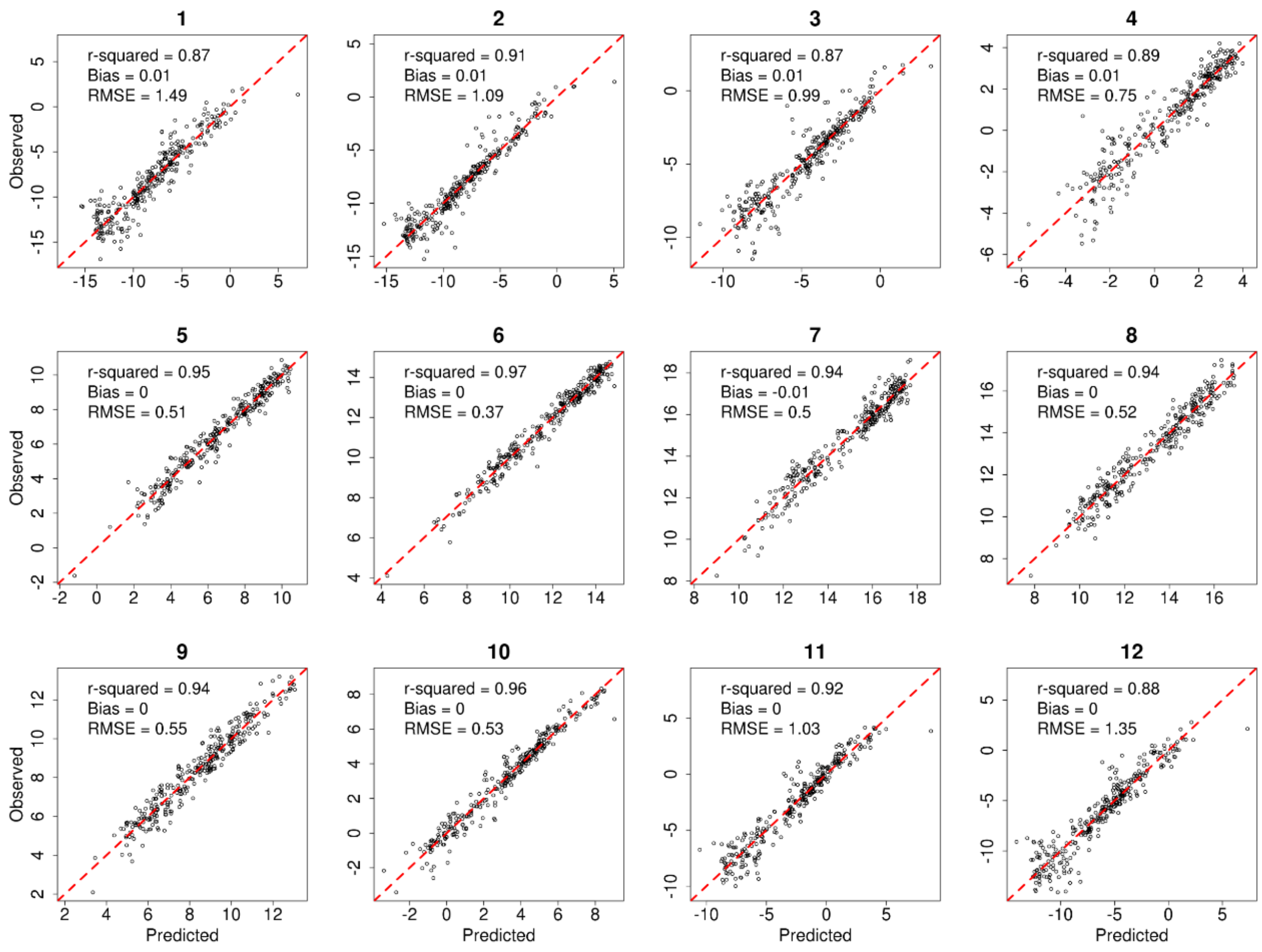

Supplementary Fig. 3S. The accuracy of the monthly average air temperature data (1981–2010, in °C). The accuracy is assessed using a leave-one-out cross-validation procedure (number of stations = 313) and is presented in terms of the adjusted r-squared, mean difference (Bias) and root mean squared error (RMSE) between the observed and predicted air temperatures. The red dashed line depicts a 1:1 line, and the subplots' title refers to a specific month (1 = January…12 = December).

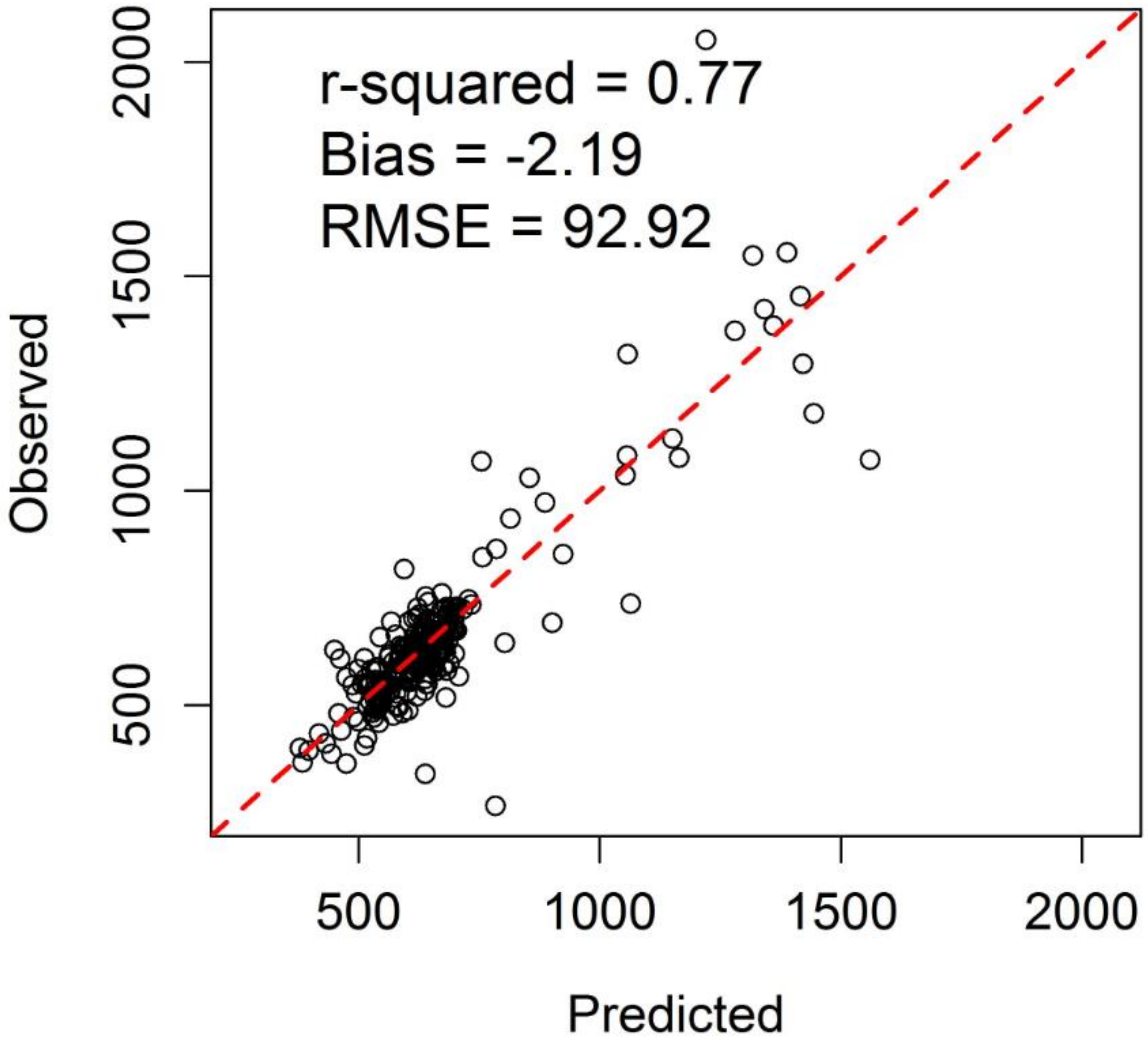

Supplementary Fig. 4S. The accuracy of the annual average precipitation data (1981–2010, in mm). The accuracy is assessed using a leave-one-out cross-validation procedure (number of stations = 343) and is presented in terms of the adjusted r-squared, mean difference (Bias) and root mean squared error (RMSE) between the observed and predicted average annual precipitation sum. The red dashed line depicts a 1:1 line.

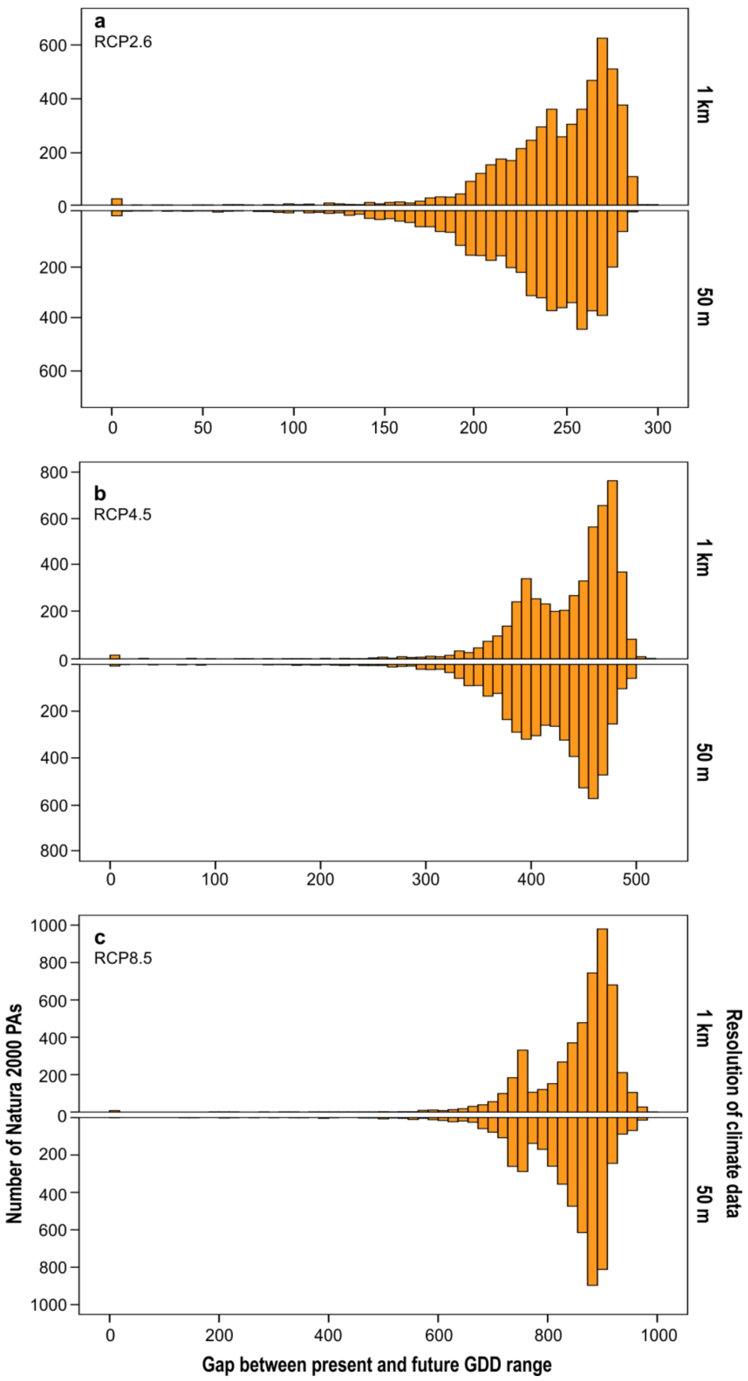

Supplementary Fig. 5S. Histograms of the gap between the present-day and the projected future range of growing degree days (GDD) within the protected areas (PAs) included in the Natura 2000 network (n = 5,068). Information is provided separately for the climate data recorded at the 1-km resolution (upper panels) and at the 50-m resolution (lower panels). Bars indicate the number of PAs showing a certain-sized gap between the current and the future climate conditions (measured as growing degree days units; °C). PAs where the compared GDD ranges overlap are all lumped together at the origin (0) of the X-axis. Contemporary within-PA ranges of GDD are compared with the corresponding future ranges measured for the three climate scenarios, RCP2.6 (a), RCP4.5 (b) and RCP8.5 (c).

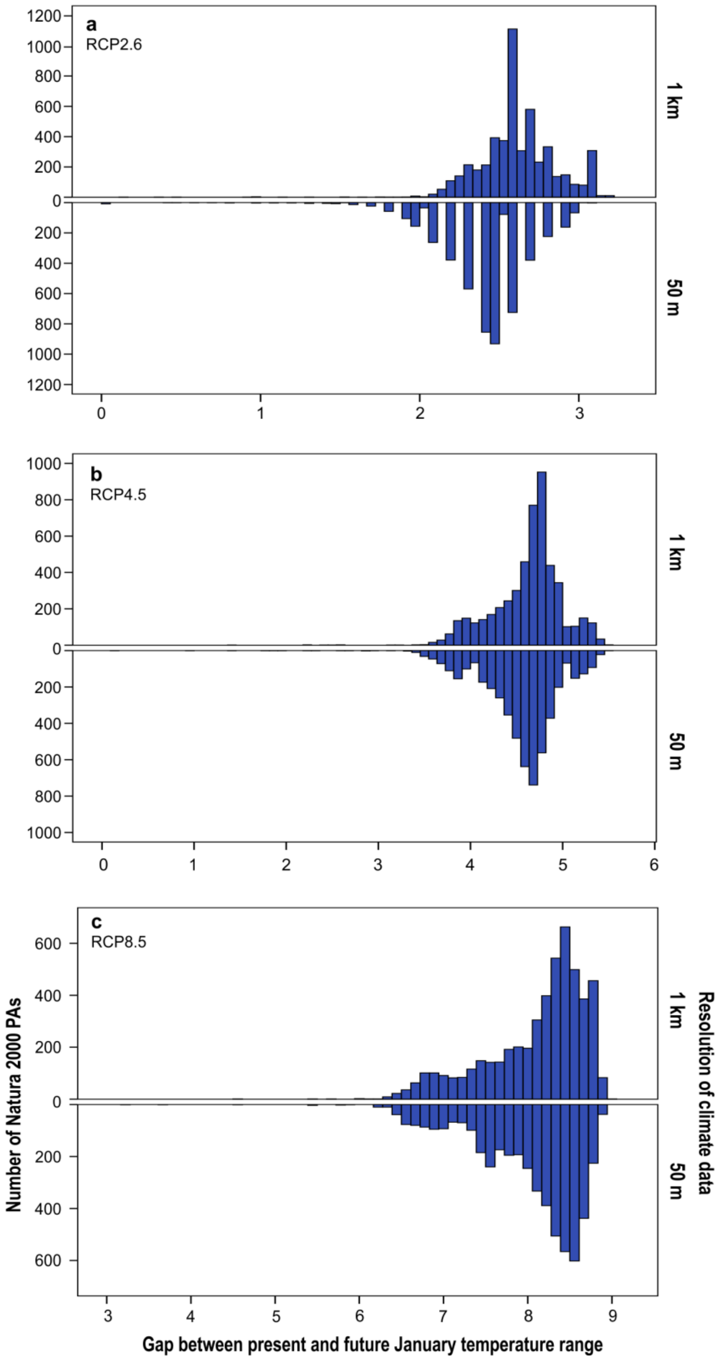

Supplementary Fig. 6S. Histograms of the gap between the present-day and the projected future range of the mean January temperature (T_{Jan}) within the protected areas (PAs) included in the Natura 2000 network (n = 5,068). Information is provided separately for the climate data recorded at the 1-km resolution (upper panels) and at the 50-m resolution (lower panels). Bars indicate the number of PAs showing a certain-sized gap between the current and the future climate conditions (measured as °C). PAs where the compared T_{Jan} ranges overlap are all lumped together at the origin (0) of the X-axis. Contemporary within-PA ranges of T_{Jan} are compared with the corresponding future ranges measured for the three climate scenarios, RCP2.6 (a), RCP4.5 (b) and RCP8.5 (c).

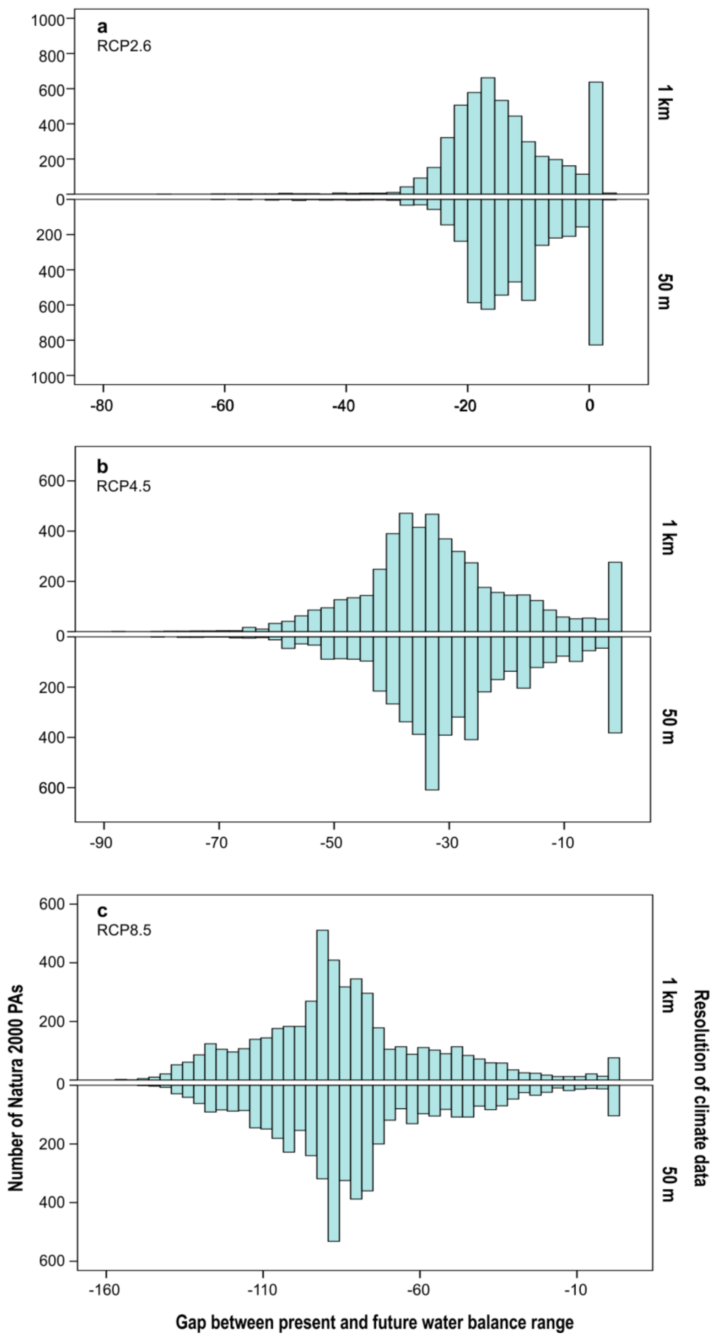

Supplementary Fig. 7S. Histograms of the gap between the present-day and the projected future range of climatic water balance (WAB) within the protected areas (PAs) included in the Natura 2000 network (n = 5,068). Information is provided separately for the climate data recorded at the 1-km resolution (upper panels) and at the 50-m resolution (lower panels). Bars indicate the number of PAs showing a certain-sized gap between the current and the future climate conditions (measured in mm). PAs where the compared WAB ranges overlap are all lumped together at the origin (0) of the X-axis, and the cases where the future range is located above the current range are excluded for the clarity of the figures. Contemporary within-PA ranges for the WAB are compared to the corresponding future ranges measured for the three climate scenarios, RCP2.6 (a), RCP4.5 (b) and RCP8.5 (c).

Supplementary Table 1S. Number of protected areas (PAs) where the climate high-velocity hotspots of the three studied climate variables overlap.

Velocity 'hotspots' (i.e. high-velocity PAs) were defined as the top 5 % of the Natura 2000 PAs according to the highest velocity values, i.e. the 253 top sites out of the total of 5,068 PAs with the highest velocities. These hotspots were first separately determined for the three considered climate variables, growing degree days with a base temperature of 5 °C (GDD), the mean January temperature (°C) and climatic water balance (mm, monthly difference between precipitation and potential evapotranspiration summed across the year) by calculating the mean of the velocities of the 50-m grid cells included in each PA. In the next step it was assessed as to how many of the PAs the velocity hotspots overlapped under the three projected future climates forced by the three Representative Concentration Pathway scenarios (RCP2.6, RCP4.5 and RCP8.5). The percentage of overlapping hotspots in relation to the maximal overlap of the 253 Natura 2000 PAs is shown in parenthesis.

**Representative Concentration Pathway**	**GDD – January temperature**	**GDD – Water balance**	**January temperature – Water balance**	**GDD - January temperature – Water balance**
RCP2.6	0 (0%)	20 (7.9%)	1 (0.4%)	0 (0%)
RCP4.5	0 (0%)	32 (12.6%)	0 (0%)	0 (0%)
RCP8.5	0 (0%)	40 (15.8%)	0 (0%)	0 (0%)

Supplementary Table 2S. Comparison of the climate-analog velocity values for the protected areas (PAs), calculated for the growing degree days with a base temperature of 5 °C (GDD) at a 50-m resolution and 1-km resolution.

The mean value and standard deviation (in parenthesis) of velocities (km/year) in the Natura 2000 PAs (n = 5,068) are shown for each climate change scenario (RCP2.6, RCP4.5, RCP8.5) separately, and divided into the three main relief regions of Finland (see Fig. 3). For these data, first the mean of the velocity values of all the grid cells embedded in a given PA was calculated. Next, each PA was assigned to one of the three main relief regions based on the PA's location (in borderline cases the assignment was based on the location of the PA's center point). The column 'Higher velocity at 50-m / 1-km resolution' shows the number of Natura 2000 PAs where the mean velocity was higher in the 50-m resolution results than in the 1-km resolution results, or vice versa. The significance of the *absolute* differences in the 50-m and the 1-km resolution velocities was tested using a paired t-test.

Representative Concentration Pathway	Main relief region	Mean 50-m velocity in PAs (*SD*)	Mean 1-km velocity in PAs (*SD*)	Higher velocity at 50-m / 1-km resolution	*t* value	*p* value
RCP2.6	1	1.98 (1.19)	2.26 (1.22)	82 / 836	18.532	<0.001
	2	1.48 (0.64)	1.73 (0.64)	189 / 2369	47.748	<0.001
	3	0.71 (0.56)	0.90 (0.62)	60 / 1532	39.831	<0.001
RCP4.5	1	3.34 (1.41)	3.58 (1.27)	116 / 802	25.611	<0.001
	2	3.30 (0.91)	3.46 (0.97)	483 / 2074	30.858	<0.001
	3	1.67 (0.93)	1.92 (1.03)	111 / 1481	33.709	<0.001
RCP8.5	1	4.80 (1.47)	4.94 (1.44)	139 / 777	14.799	<0.001
	2	6.12 (0.89)	6.63 (0.79)	105 / 2452	77.071	<0.001
	3	3.78 (1.62)	4.19 (1.88)	120 / 1472	38.094	<0.001

Main relief region: 1 – Flatland with mostly even terrain; 2 – Hilly, undulating terrain varying in height; 3 – rugged terrain with deep valleys or high steeply sloped fell areas.

Degrees of freedom: Main relief region 1 – 917; 2 – 2,557; 3 – 1,591

Supplementary Table 3S. Number of PAs belonging to the top 5 % (253 out of the total of 5,068) of PAs showing the largest *relative* difference between two velocity values for GDD. The table provides the data on the largest *relative* difference between the 50-m resolution and the 1-km resolution climate data based on the velocity values measured for GDD. The division of the top 5 % of the Natura 2000 PAs with the largest differences in the three main relief region categories of Finland is shown. The top PAs with the largest differences between the two velocity values are predominantly located in rugged terrain with notable variations in elevation (relief region 3). The expected values (*in italics*) for the three main relief regions are measured based on the total number of PAs situated in each of the three regions. The top 5 % velocity differences are shown for each of the RCPs separately, and in all the PAs with the top differences the velocity values for the 50-m resolution are smaller than the velocity values for the 1-km resolution.

**Representative Concentration Pathway**	**Main relief region 1 (*18.1 %*)**	**Main relief region 2 (*50.5 %*)**	**Main relief region 3 (*31.4 %*)**
RCP2.6	60 (23.7 %)	49 (19.4 %)	144 (56.9 %)
RCP4.5	70 (22.7%)	14 (5.5 %)	169 (66.8 %)
RCP8.5	40 (15.8 %)	40 (15.8 %)	173 (68.4 %)

Main relief region: 1 = Mostly even terrain, often flatlands, including 918 PAs; 2 = undulating hilly terrain, including 2,558 PAs; 3 = Rugged terrain, often with fells and gorges, including 1,592 PAs.

Supplementary Table 4S. Results of generalized linear models (GLM) linking the main relief region, size of the protected areas (PA), within-PA elevation range and climate scenario to the *relative* difference between the two GDD velocity values, and the effect size for the first three predictors.

Here, we developed a full GLM model to test the importance of the relief region, and as a comparison, the size of the Natura 2000 PA, within-PA elevation range and climate scenario, to explain the relative difference between the two velocity values. All four variables were entered simultaneously in the full GLM model, in which the relief region was treated as an ordinal variable with three levels, the size of the Natura 2000 PA and within-PA elevation range both as log-transformed continuous variables, and the climate scenario (RCP2.6, RCP4.5 and RCP8.5) as a categorical factor. The explanatory power and statistical significance were assessed based on the t values and p-values derived from the full GLM model, and further examined for the three variables of main interest (the relief region, size of the PA and within-PA elevation range) by the effect size of a predictor. This was determined based on the range between their predicted minimum and maximum values over the observation data while controlling for the influence of other predictors by fixing them at their mean values (Nakagawa and Cuthill 2007).

Dependent variable: Relative difference between the two velocity values

Source of variation	Estimate	Std. Error	t value	p-value	Effect size
(Intercept)	17.88	1.2768	14.008	<0.001	-
log(Area)	1.40	0.2009	6.966	<0.001	3.6129
relief region	8.63	0.4474	19.298	<0.001	17.268
log(1+Range)	-3.57	0.3678	-9.712	<0.001	24.714
RCP2	-20.0985	0.7209	-27.878	<0.001	
RCP3	-23.1340	0.7209	-32.089	<0.001	

glm_full <- glm(formula = Relative difference ~ log(Area of Natura 2000 PA) + relief region + log(1 + range) + RCP, family = Gaussian)

Null deviance: 22134716 on 15203 degrees of freedom

Residual deviance: 20262318 on 15198 degrees of freedom

References

Nakagawa, S. & Cuthill, I. C. 2007. Effect size, confidence interval and statistical significance: a practical guide for biologists. Biological Reviews **82**, 591-605.

Supplementary Table 5S. Number of protected areas (PAs) where the present-day and the projected future range of a given climate variable overlap.

The statistics are given for the two different climate data resolutions employed in the study, the 50-m resolution and the 1-km resolution. The comparison indicates that fine-grained topoclimate data shows a higher frequency of overlapping between the present-day and the future climate ranges in the studied PAs than the 1-km climate data.

	GDD		Mean January temperature		Water balance	
**Representative Concentration Pathway**	**50-m resolution**	**1-km resolution**	**50-m resolution**	**1-km resolution**	**50-m resolution**	**1-km resolution**
RCP2.6	17	13	7	0	823	563
RCP4.5	7	5	0	0	342	254
RCP8.5	1	1	0	0	103	76

Supplementary Table 6S. Number of protected areas (PAs) where the gap between the present-day and the projected future within-PA range of a given climate variable is larger at the 50-m resolution or 1-km resolution.

For the GDD and mean January temperature, the trend is always unidirectional in that the future range is either overlapping with the current range or fully separated above it with no overlapping parts in the ranges. For the climatic water balance, the future range is overlapping with the current range or fully separated from the current range either above or (in most cases) below it. The gap between the present-day and the projected future within-PA range is typically bigger in the 1-km resolution climate data than in the 50-m resolution climate data.

	No of cases with larger gap at 50-m resolution / 1-km resolution			
**Representative Concentration Pathway**	**GDD**	**Mean January temperature**	**Water balance; future range above the current range**	**Water balance; future range below the current range**
RCP2.6	895 / 4,173	212 / 4,856	9 / 61	805 / 3,628
RCP4.5	899 / 4,150	1,199 / 3,593	0 / 21	96 / 4,681
RCP8.5	918 / 4,169	1,199 / 3,593	0 / 4	25 / 4,957
